# Factors Associated with Hyperpolypharmacy and Complex Medication Regimens in Kidney Transplant Recipients

**DOI:** 10.3390/jcm13133716

**Published:** 2024-06-26

**Authors:** Armin Atić, Jasmina Matijašević Škerlj, Ivana Jurić, Lea Katalinić, Vesna Furić Čunko, Marina Kljajić, Zoran Sabljić, Bojan Jelaković, Nikolina Bašić-Jukić

**Affiliations:** 1 Division of Nephrology, Arterial Hypertension, Dialysis and Transplantation, University Hospital Center Zagreb, Kispaticeva 12, 10000 Zagreb, Croatia; 2 Hospital Pharmacy, University Hospital Center Zagreb, 10000 Zagreb, Croatia

**Keywords:** kidney transplantation, polypharmacy, multiple chronic conditions

## Abstract

**Background**: Kidney transplantation is considered the best modality for renal replacement therapy. The use of immunosuppressive therapy and pre-existing and newly developed comorbidities predispose these patients to the use of a large number of medications. (Hyper)polypharmacy is associated with worse adherence and negative outcomes. This study aims to explore the factors correlated with hyperpolypharmacy and complex medication regimens in kidney transplant recipients. **Methods**: This is a cross-sectional study of outpatient kidney transplant recipients. Collected data include demographic data, complete chronic medication lists, medical history, and graft function. Linear and logistic regression were used to identify factors associated with hyperpolypharmacy and complex medication regimens. Medication regimen complexity was quantified by the Medication Regimen Complexity Index (MRCI). **Results**: Overall, 224 kidney transplant recipients were included, with an average time since transplantation of 8 years. Hyperpolypharmacy was present in more than two-thirds of patients; the average number of different medications was 12; and the mean MRCI score was 21.4, ranging from 6 to 50. Hypertension was almost universally present, while other frequently prescribed medication groups were hypolipemics, medication for bone-mineral metabolism disorders, gout, and antihyperglycemics. **Conclusions**: Factors independently associated with hyperpolypharmacy and complex medication regimens were found to be age and graft function. Studies investigating interventions aimed at reducing medication complexity and increasing adherence should focus on older patients with worse graft function.

## 1. Introduction

Kidney transplantation is the preferred method for renal replacement therapy (RRT). providing patients with significantly improved quality of life, reduced cardiovascular risk, and improved survival over other RRT modalities [1,2]. After kidney transplantation, however, patients are required to use several immunosuppressive medications. The most commonly used immunosuppressives are calcineurin inhibitors (CNIs), corticosteroids, mTOR inhibitors, and mycophenolic acid. Immunosuppressive medications, particularly CNIs, significantly influence metabolic processes and result in derangements such as impaired glucose tolerance, hyperlipidemia, and increased blood pressure. These side effects frequently require treatment and the introduction of additional medications, and kidney transplantation has been shown to increase both the overall pill burden and medication regimen complexity when compared to pre-transplantation medication [3]. The presence of several chronic conditions, termed multimorbidity, is highly prevalent across the chronic kidney disease (CKD) spectrum, and kidney transplant recipients are no exception [4]. Importantly, kidney transplant recipients do have a decreased multimorbidity prevalence than patients with advanced CKD; the prevalence of multimorbidity has been reported to be 80% in kidney transplant recipients (KTRs), almost half of them having complex multimorbidity [4]. Most prevalent comorbidities include hypertension; disorders of the musculoskeletal system, including bone-mineral disorders; cardiovascular diseases; and diabetes. Such a high prevalence of comorbidities necessitates the use of one or more medications to treat these conditions. Polypharmacy is usually defined as the concomitant use of 5–9 medications, while hyperpolypharmacy pertains to the use of 10 or more medications. Both are associated with a lower quality of life and, in older patients, functional decline, falls, and cognitive impairment [5,6]. Polypharmacy in KTRs may pose additional risks of adverse drug effects, drug interactions, and non-adherence to medication, as KTRs all have impaired renal function and additionally need to keep immunosuppressant concentrations within tight windows. Furthermore, polypharmacy has been associated with a reduced intake of fiber and several types of vitamins and minerals, as well as an increased intake of carbohydrates and sodium [5,7]. Drug–drug interactions pose a particular challenge in kidney transplant recipients, as immunosuppressive medication is the most frequent cause of such interactions [8,9]. Medication complexity, however, is not only affected by the number of different medications but also by the number of daily doses, the route of administration, dose variability, and other factors associated with more complex medication intake. The Medication Regimen Complexity Index (MRCI) is a tool developed and validated for quantifying medication complexity [10,11]. It takes into account numeric variables, such as the number of drugs and the number of doses, as well as additional variables such as ease of use, specific intake instructions, and dose variability in the overall assessment. Higher medication complexity assessed by MRCI (but not polypharmacy) was associated with higher mortality in older people in some studies [12,13]. Another study of older patients with heart failure failed to identify medication complexity as a risk factor for rehospitalization or mortality [14].

(Hyper)polypharmacy is considered a modifiable risk factor; however, in kidney transplant recipients, it is often unavoidable. Understanding its consequences, as well as potential risk factors, may help in early identification of patients likely to be prescribed large numbers of drugs or complex medication regimens, with the goal of patient preparation, as well as education, in order to promote medication adherence and reduce the unwanted health effects associated with hyperpolypharmacy.

This study aims to identify risk factors associated with hyperpolypharmacy and complex medication regimens, with a focus on non-modifiable factors associated with previous renal replacement therapy (RRT) and patient-specific factors.

## 2. Materials and Methods

This is a cross-sectional study including outpatient kidney transplant recipients who visited the transplantation outpatient clinic during the month of June 2023. Data were collected by two independent researchers from the hospital information system (BIS, IN2 group, Zagreb, Croatia). Collected data were sex, age, weight, height, date of transplantation, pre-transplantation renal replacement therapy type and duration, complete medication lists, and relevant laboratory results. Height and weight were collected only if recorded at the time of patient visit. We excluded patients who were transplanted within the past year due to potential interference of universal prophylaxis drugs and patients with unavailable full chronic medication lists. Medication complexity was analyzed using the MRCI. The MRCI score was calculated by two researchers using a data capture and coding tool available online, per the instructions of the developers. Data collection and the calculation of MRCI scores were overseen by a senior investigator. Hyperpolypharmacy was defined as the concomitant use of 10 or more drugs. There is no uniform cutoff MRCI score for complex medication regimens; research suggests population-specific cutoff values. In our study, we defined complex medication regimens as those with MRCI > 25.8, a mean value identified in a previous report [15].

Categorical variables are presented as counts and percentages. Comparison among groups was performed with the chi-squared test. The Kolmogorov–Smirnov test was used to test for the normality of distribution of continuous variables. Continuous variables are presented as means and standard deviations or medians and interquartile ranges (IQR) where appropriate. For comparison of continuous and ordinal variables, Student’s *t*-test or the Mann–Whitney test was used. To determine the predictors of hyperpolypharmacy and MRCI score, univariate and multiple logistic regression were used. Statistical analysis was performed using IBM SPSS software, version 26.0 (IBM Corp. Released 2019. IBM SPSS Statistics for Windows, Version 26.0. Armonk, IL, USA).

Data regarding donor type, RRT prior to transplantation, and dialysis vintage were missing for patients transplanted prior to the adoption of the current hospital information system in 2011. Paper charts and old medical registries were searched for data; for patients for whom no data were available, variables were marked as missing. Data regarding height and weight were missing for slightly older patients and for patients who were transplanted significantly earlier than for patients with present values (9 vs. 18 years). Patients with missing BMIs had lower MRCI scores (17 vs. 22), which is a potential source of bias. Missing data were excluded from the statistical analysis (case-wise), and the counts of missing data for each variable are presented in Supplementary Table S1.

## 3. Results

### 3.1. Cohort Characteristics

Overall, 258 patient visits were reviewed, and after exclusion of 34 patients (21 due to incomplete medication lists and 13 <1 year since transplantation), 224 patients were included in the study. The included patients were more frequently male (62.1), and the average age was 56 years, with a an average time since transplantation of 10.6 years. The most frequent method of RRT prior to transplantation was hemodialysis (71.9%), while 15.9% of patients were on peritoneal dialysis and 7% were on both RRT modalities. Dialysis vintage was, on average, 3.5 years; for patients with prior RRT, the lowest dialysis vintage was 2 months, and the highest was 25 years. The majority of patients had an abnormal body mass index (BMI) (61.5%), with 38% of patients classified as overweight and 22.4% as obese. Graft function was well preserved in most patients, with a mean eGFR of 53 mL/min/1.73 m^2^, with only 13.2% of patients with an eGFR below 30. 

### 3.2. Medication Regimen Characteristics

On average, the patients used 12 different medications (lowest 2, highest 21), with an average MRCI score of 21.3. In the studied population, only six patients (3%) were not classified as polypharmacy, while more than two-thirds (71%) were classified as hyperpolypharmacy. The MRCI score varied widely, with the simplest regimen rated at 6 and the most complex at 50. Slightly more than half (55.8%) of patients had a treatment regimen simpler than the mean, as rated by the MRCI score. 

The majority of patients had calcineurin inhibitors (CNIs) in their immunosuppressive regimen, 72% of whom were using tacrolimus, which was combined chiefly with mycophenolic acid or with mTOR inhibitors in 10% of patients. Overall, one-fifth of patients were using mTOR inhibitors, while azathioprine was in the immunosuppressive regimen of 6% of included patients. Twelve patients (5.3%) were on steroid-free regimens. Almost all patients (92%) were treated for arterial hypertension, with calcium channel blockers (CCBs) and beta blockers as the most commonly used agents, followed by centrally acting antihypertensives, RAAS inhibitors, and alpha-1 antagonists. Diuretic use was also highly prevalent (70%), with the vast majority being loop diuretics and thiazide/thiazide-like diuretics and mineralocorticoid antagonists prescribed in 5% of patients each. One-fifth of patients were treated for diabetes mellitus, chiefly with insulin, DPP-4 inhibitors, metformin, and glinides (Supplementary Table S1). More than half of patients were prescribed statin therapy, with only a small percentage of patients using ezetimibe. Other frequently prevalent medications were xanthine oxidase inhibitors (46% of patients) and medications used to treat bone-mineral metabolism disorders (49%) (Supplementary Table S2). 

Patients using fewer than 10 drugs were younger by a mean of 8 years; were more frequently female; had their current graft for a longer period, with the graft exhibiting better function as predicted by the eGFR; and had a lower BMI. Dialysis vintage, type of renal replacement therapy before transplantation, and donor type did not differ significantly among groups (Table 1). Similarly, patients with a less complex MRCI score (cutoff of 25.8) were younger (57 vs. 65 years, *p* < 0.001) and had better graft function (eGFR 55 vs. 39, *p* < 0.001); however, there were no statistically significant differences in time since transplantation, dialysis vintage, or BMI. Additionally, no statistically significant differences among groups were found for prior RRT type, donor type, or sex.

### 3.3. Factors Associated with Complex Medication Regimens

Patient characteristics stratified according to the presence of hyperpolypharmacy are presented in Table 1. Factors univariately associated with hyperpolypharmacy were age (OR 1.048, 95% CI 1.024–1.072), female sex (OR 1.939, 95% CI 1.078–3.49), BMI (OR 1.107, 95% CI 1.016–1.206), time since transplantation (OR 0.943, 95%CI 0.904–0.984), and eGFR (OR 0.966, 95% CI 0.951–0.98). In the multivariable logistic regression model including all the variables identified by the univariate analysis and including dialysis vintage and time since transplantation, only eGFR and age were recognized as mutually independent predictors of hyperpolypharmacy. 

Lower graft function assessed by the eGFR was independently associated with higher odds of hyperpolypharmacy (OR 0.969, 95% CI 0.950–0.989), as was increased age (OR 1.045, 95% CI 1.008–1.084) and a higher BMI (OR 1.163, 95% CI 1.040–1.300).

Patient characteristics stratified according to the high MRCI score are presented in Table 2. Factors univariately associated with a high MRCI score were age (OR 1.052, 95%CI 1.023–1.081) and eGFR (OR 0.96, 95% CI 0.943–0.978). In the multivariable model including variables with univariate associations and those selected by sound clinical judgement (donor type, dialysis vintage, time since transplantation, and gender), high MRCI was predicted by donor type, eGFR, and age. Having a living donor was associated with significantly lower odds of a complex MRCI score status (OR 0.149, 95% CI 0.024–0.933). Of the other variables in the model, increased age (OR 1.057, 95% CI 1.017–1.098) and a lower eGFR (OR 0.967, 95% CI 0.945–0.988) were associated with higher odds of a high MRCI score.

## 4. Discussion

The results of this retrospective study of 224 included kidney transplant recipients show a high prevalence of hyperpolypharmacy and complex medication regimens. Factors independently associated with both hyperpolypharmacy and complex medication regimens were age and graft function. Additionally, patients with a higher BMI had higher odds of complex medication regimens but not hyperpolypharmacy.

In the studied cohort, patients with longer-functioning grafts, interestingly, had lower rates of hyperpolypharmacy but not lower MRCI scores; however, multivariable regression analysis did not find an independent association of time since transplantation with hyperpolypharmacy or medication regimen complexity. 

Adherence to the prescribed medication regimens is critical in the treatment of kidney transplant recipients. Non-adherence in kidney transplant recipients has been associated with increased rates of graft rejection and death [16]. Using less complex medication regimens has led to improved adherence, and recognition of factors predisposing patients to necessary complex medication regimens may lead to improved patient collaboration and, ultimately, adherence [17,18]. Previous studies that have evaluated medication complexity have shown a high level of contribution from transplantation-related drugs, and transplantation itself has been shown to increase medication complexity more than any other chronic state, such as diabetes, hypertension, HIV, or geriatric depression [19].

The average number of medications used was higher than in two previous reports; however, the overall MRCI score was lower in our cohort [3,15]. These studies, however, evaluated patients up to 12 months after transplantation and did not report graft function at the studied time points. The cohorts in a study by Marienne and our study were similar in terms of age, sex, and prevalence of diabetes and dyslipidemia, but the prevalence of hypertension was significantly higher in our study. The large proportion of patients with good graft function may explain the lower MRCI score in our cohort. Other possible explanations for the differences include the possible use of different formulations of drugs, such as tacrolimus, which has immediate-release forms that require twice-daily dosing and slow- and extended-release formulations that are taken once daily [20].

Peritoneal dialysis has several advantages over hemodialysis, including both pre- and post-transplantation outcomes such as reduced cardiovascular risks, lower overall mortality on dialysis, and a lower incidence of delayed graft function [21]. Interestingly, our results show that there is no difference between RRT types regarding post-transplantation medication regimen complexity or the occurrence of hyperpolypharmacy. Additionally, although a longer dialysis vintage is associated with poorer outcomes post-transplantation, our study shows no effects of dialysis vintage on medication regimen complexity [22]. There are no previous studies evaluating the effects of these variables. A longer time since transplantation was found not to predict more complex medication regimens after corrections for graft function and age. 

The use of a living donor (but not pre-emptive transplantation) was associated with lower-complexity medication regimens. Usually, pre-emptive transplantation occurs in the setting of a living donor, most frequently in the pediatric setting, implying that these patients are usually younger, have fewer comorbidities, and have spent no time on other RRT modalities. Our multivariable analysis showed beneficial effects of a living donor on medication regimen complexity, independent of age and other factors. However, the overall number of pre-emptively transplanted patients (i.e., those who were never treated with dialysis) and patients who were transplanted from a living donor were low, and this study is underpowered to determine that association.

Despite the difference in prevalence of hyperpolypharmacy and complex medication regimens in the studied population, predictive factors were virtually the same. Of the studied factors, only BMI was shown to be a factor for hyperpolypharmacy but not complex medication regimens. Although obesity has been associated with polypharmacy in other studies in the general population, from our data, we were unable to determine the reasons for the lack of association with the MRCI score [23]. We have not explored the effect of using other cutoff values for a high MRCI score and its effect on determining predictors.

This study has several limitations. The first is the limitation innate to the retrospective design. Other limitations include the lack of correction for other potential confounding factors, such as the presence of comorbidities and their severity. However, we believe that the prescription of more medication may serve as a proxy for the presence of comorbidities. Furthermore, our study did not assess the adherence of patients to medication, which may, in some, cases lead to the prescription of more drugs (e.g., for treatment of hypertension in non-adherent patients). Another highlight of this study and a possible limitation is the lack of a definition of complex medication regimens. We opted for the use of a mean value reported in previous studies, which was higher than the mean value in our cohort.

Our results indicate that factors associated with increased morbidity and mortality, such as RRT type and length, and other factors such as time since transplantation or type of immunosuppression do not affect chronic medication complexity in renal transplant recipients. The correlations of age and graft function may indicate that revision of medication lists and other adherence-promoting measures may be best suited for older patients and patients with worsening graft function. Unlike in other populations, it is unknown whether the MRCI score is related to increased mortality and whether reducing medication regimen complexity can lead to beneficial outcomes in this population. Further studies are needed to investigate whether those correlations exist in KTRs. Additional studies regarding medication adherence and medication complexity in KTRs are needed.

## 5. Conclusions

Hyperpolypharmacy and complex medication regimens are highly prevalent in kidney transplant recipients. The main factors associated with hyperpolypharmacy and complex medication regimens are age and graft function. Studies investigating interventions aimed at reducing medication complexity and increasing adherence should consider older patients and those with worsening graft function.

## Figures and Tables

**Table 1:** Comparison of groups according to hyperpolypharmacy.

Characteristic	Hyperpolypharmacy (N = 159)	<10 Drugs (N = 65)	
Male sex, N (%)	106 (67%)	33 (51%)	*p* = 0.02
Age, median, IQR *	60 (50–68)	54 (38–61)	*p* < 0.001
RRT type prior to transplantation			*p* = 0.3
Hemodialysis	110	33	
PD	22	9	
Both	9	5	
No RRT	6	5	
Dialysis vintage, years, median (IQR) *	2.9 (1.5–4.6)	2.5 (0.8–4)	*p* = 0.2
Time since transplantation, years *	9 (7–18)	11 (7–12.5)	*p* = 0.017
eGFR, mL/min/1.73 m^2^, median (IQR) *	45 (36–61)	63 (49–79)	*p* < 0.001
BMI	27 (4.3)	25.3 (3.9)	*p* = 0.019
Cadaveric donor	151	52	*p* = 0.075
CNI/mTOR/both			*p* = 0.048
CNI	126	48	
mTOR	18	9	
Both	15	5	
Neither	0	3	
Type of CNI			*p* = 0.157
Cyclosporine	37	19	
Tacrolimus	104	34	
Azathioprine	6	7	*p* = 0.051
MMF	137	51	*p* = 0.112

* Mann–Whitney U test; PD—peritoneal dialysis; RRT—renal replacement therapy; eGFR—estimated glomerular filtration rate; BMI—body mass index; CNI—calcineurin inhibitor; mTORi—mammalian target of rapamycin inhibitor; MMF—mycophenolate mofetil.

**Table 2:** Comparison of groups according to complex medication regimens (cutoff MRCI score of 25.8).

Characteristic	MRCI Score < 25.8 (N = 171)	MRCI Score > 25.8 (N = 53)	
Male sex, N (%)	106 (62%)	33 (62%)	*p* = 0.9
Age, median, IQR *	57 (45–64)	65 (55–72)	*p* < 0.001
RRT type prior to transplantation			*p* = 0.561
Hemodialysis	104	39	
PD	25	6	
Both	12	2	
No RRT	9	2	
Dialysis vintage, years, median (IQR) *	3 (1.3–4.8)	2.3 (1.5–4)	*p* = 0.2
Time since transplantation, years *	9 (5–14)	11 (6–14)	*p* = 0.017
eGFR, mL/min/1.73 m^2^, median (IQR) *	55 (45–64)	39 (29–55)	*p* < 0.001
BMI	26.5 (4.4)	26.7 (4.1)	*p* = 0.785
Cadaveric donor	154	49	*p* = 0.826
CNI/mTOR/both			*p* = 0.461
CNI	135	39	
mTOR	20	7	
Both	13	7	
Neither	3	0	
Type of CNI			*p* = 0.963
Cyclosporine	42	14	
Tacrolimus	106	32	
Azathioprine	4	2	*p* = 0.981
MMF	93	44	*p* = 0.417

* Mann–Whitney U test; PD—peritoneal dialysis; RRT—renal replacement therapy; eGFR—estimated glomerular filtration rate; BMI—body mass index; CNI—calcineurin inhibitor; mTORi—mammalian target of rapamycin inhibitor; MMF—mycophenolate mofetil.

## Data Availability

The raw data supporting the conclusions of this article will be made available by the authors upon request.

## Supplementary Materials

The following supporting information can be downloaded at: https://www.mdpi.com/article/10.3390/jcm13133716/s1, Table S1: Patient characteristics; Table S2: Chronic medication characteristics.
