# Supplementary material for: Factors Associated with Hyperpolypharmacy and Complex Medication Regimens in Kidney Transplant Recipients
Source: J Clin Med. 2024 Jun 26;13(13):3716. doi: 10.3390/jcm13133716 (PMC11242636; doi:10.3390/jcm13133716)
Supplement: Supplementary file 1 [file jcm-13-03716-s001.zip › jcm-3071603-supplementary.pdf]

Table S1 – Patient characteristics		
Variable		Missing values
Age, median (IQR)	58 (47-66)	0
Male sex, N (%)	139 (62,1)	0
BMI, mean (SD)	26,6 (4,3)	50
Underweight	2 (1.1)	
Healthy weight	67 (38.5)	
Overweight	66 (38)	
Obese (first class)	30 (17.2)	
Obese (second class)	9 (5.2)	
RRT type before transplantation, N (%)		26
Hemodialysis	143 (71.9)	
Peritoneal dialysis	31 (15.6)	
Both	13 (7)	
No RRT	11 (5.5)	
Dialysis vintage, years, median (IQR)	2 (1-4)	30
Cadaveric donor, N (%)	203 (93.1)	6
Time since transplantation, years, median (IQR)	8 (5-12)	0
eGFR (ml/min/1.73m^2), median (IQR)	48 (37-63)	0
< 15, N (%)	3 (1.3%)	
15-30, N (%)	28 (12.5)	
30-45, N (%)	63 (28.1)	
45-60, N (%)	54 (24.1)	
> 60, N (%)	76 (33.9)	
PD – peritoneal dialysis; RRT – renal replacement therapy; eGFR – estimated glomerular filtration rate; BMI – body mass index; CNI – calcineurin inhibitor; mTORi – mammalian target of rapamycin inhibitor; MMF – mycophenolate mofetil		

Supplementary Table S2 – Chronic medication characteristics	
Medication number, mean (SD)	12 (4)
MRCI score, mean (SD)	21.4 (7.7)
Immunosupression	
CNI	195 (87)
Tacrolimus	140 (62)
Cyclosporine	56 (25)
mTOR	46 (20.5)
MMF	188 (84)
Azathioprine	13 (6)
Antihypertensive drugs	205 (91.5)
ACEi/ARB	83 (37)
Beta blockers	145 (65)
CCI	146 (65)
Alpha antagonists	68 (30)
Centrally acting	104 (46)
Nitrates	10 (5)
Diuretics	158 (70.6)
Loop diuretics	147 (65.9)
Thiazide/TLD	11 (4.9)
MRA	9 (4)
Diabetes drugs	45 (20.1)
Metformin	11 (4.9)
Glinides	10 (4.5)
Sulfonylurea	3 (1.3)
GLP-1RA	2 (0.9)
Thiazolidinediones	3 (1.3)
DPP-4 inhibitors	12 (5.4)
SGLT2i	5 (2.3)
Insulin	19 (8.5)
Antilipemic drugs	122 (54.4)
Statins	119 (53.1)
Ezetimibe	6 (2.7)
Uric acid drugs	103 (46)
Allopurinol	99 (44.2)
Febuxostat	3 (1.3)
Bone-mineral metabolism drugs	109 (48.7)
Phosphate binders	14 (6.3)
Vitamin D analogues	87 (38.8)
Calcimimetics	21 (9.4)
Bisphosphonates	2 (0.9)
MRCI – Medication Regimen Complexity Index; CNI – Calcineurin inhibitor; mTOR – mammalian target of rapamycin; MMF – Mycophenolate mofetil; ACEi – angiotensin converting enzyme inhibitor; ARB –	

angiotensinogen receptor blocker; CCI – calcium channel blocker; TLD – Thiazide-like diuretic; MRA – 2
mineralocorticoid receptor antagonists; GLP-1 RA – glucagon like peptide-1 receptor agonist; DPP-4 - Dipeptidyl peptidase-4 inhibitor; SGLT2i – sodium-glucose cotransporter-2 inhibitors
